# Unique sequence features of the Human Adenovirus 31 complete genomic sequence are conserved in clinical isolates

**DOI:** 10.1186/1471-2164-10-557

**Published:** 2009-11-25

**Authors:** Soeren Hofmayer, Ijad Madisch, Sebastian Darr, Fabienne Rehren, Albert Heim

**Affiliations:** 1 Institut für Virologie, Medizinische Hochschule Hannover, Hannover, Germany; 2 Current address: Department of Radiology, Massachusetts General Hospital Harvard Medical School 100 Charles River Plaza, Suite 400 Boston, Massachusetts 02114, USA

## Abstract

**Background:**

Human adenoviruses (HAdV) are causing a broad spectrum of diseases. One of the most severe forms of adenovirus infection is a disseminated disease resulting in significant morbidity and mortality. Several reports in recent years have identified HAdV-31 from species A (HAdV-A31) as a cause of disseminated disease in children following haematopoetic stem cell transplantation (hSCT) and liver transplantation. We sequenced and analyzed the complete genome of the HAdV-A31 prototype strain to uncover unique sequence motifs associated with its high virulence. Moreover, we sequenced coding regions known to be essential for tropism and virulence (early transcription units E1A, E3, E4, the fiber knob and the penton base) of HAdV-A31 clinical isolates from patients with disseminated disease.

**Results:**

The genome size of HAdV-A31 is 33763 base pairs (bp) in length with a GC content of 46.36%. Nucleotide alignment to the closely related HAdV-A12 revealed an overall homology of 84.2%. The genome organization into early, intermediate and late regions is similar to HAdV-A12. Sequence analysis of the prototype strain showed unique sequence features such as an immunoglobulin-like domain in the species A specific gene product E3 CR1 beta and a potentially integrin binding RGD motif in the C-terminal region of the protein IX. These features were conserved in all analyzed clinical isolates. Overall, amino acid sequences of clinical isolates were highly conserved compared to the prototype (99.2 to 100%), but a synonymous/non synonymous ratio (S/N) of 2.36 in E3 CR1 beta suggested positive selection.

**Conclusion:**

Unique sequence features of HAdV-A31 may enhance its ability to escape the host's immune surveillance and may facilitate a promiscuous tropism for various tissues. Moderate evolution of clinical isolates did not indicate the emergence of new HAdV-A31 subtypes in the recent years.

## Background

Adenoviridae are non-enveloped, double-stranded DNA viruses with an icosahedral capsid [1]. Human Adenoviruses (HAdV) belong to the genus Mastadenovirus and are classified into six species (HAdV-A to HAdV-F) that were defined historically as subgenera on the basis of hemagglutination properties [2,3]. Subsequently, oncogenic properties in rodents and DNA homology were also used to define the subgenus (species) [1]. Recently a new strain of HAdV was discovered and has been classified as HAdV-52, representing a putative new species G [4].

Human adenoviruses have long been recognized as pathogens causing a broad spectrum of different diseases depending on the type-related organotropism and virulence. For example, infections of the upper respiratory tract are caused by HAdV-C1, -C2, -C5, -B3, and -B7 [5,6], the more dangerous infections of the lower respiratory tract mainly by HAdV-B3, -B7, -B21, and -E4 [7-9]. The types HAdV-D8, -D19, and -D37 are closely associated with severe epidemic keratoconjunctivitis. Gastroenteritis and diarrhoea are caused by the enteric adenoviruses HAdV-F40,-F41 and HAdV-A31, which are frequently found in infants and children.

Moreover, immunocompromised patients can develop a sepsis-like, disseminated adenovirus syndrome that is associated with high levels of immunosuppression (for example, lymphocyte counts <300/μl) as a crucial risk factor [10]. A wide range of organs can be affected and an effective antiviral therapy is not yet available. Consequently, mortality rates of up to 60% were reported [10-12]. Disseminated disease is mainly caused by species C adenoviruses. However, in recent decades, HAdV-A31 has been increasingly reported as a etiologic agent for dissemination in immunosuppressed children following allogenic haematopoietic stem cell transplantation [10,13-15].

One of the essential sequence features for HAdV types causing dissemination may be the viral RGD (integrin binding) motif of the penton base protein, because HAdV-F types lacking the RGD motif have never caused a disseminated infection (with exception of a single case report) in spite of their high prevalence [16-18]. In addition, recent studies have shown that the binding of blood coagulation factor (F) X to the HAdV-C5 hexon protein facilitates infection of the liver and could also foster virus dissemination [19]. Similar to HAdV-C5, F IX binding may promote HAdV-A31 infection of epithelial cells [20].

The outstanding clinical relevance of HAdV-A31 is also documented by its significant association with immunosuppressed patients in comparison to immunocompetent patients [16]. This high incidence may be explained by reactivations of latent (or persisting) HAdV infections, which have been recently described for species C HAdV [21]. A similar mechanism may be suspected for HAdV-A31 although any conclusive data on its persistence is still lacking. So far, genetic analysis of HAdV-A31 strains isolated from hSCT patients showed significant differences even in the same clinical centre, suggesting reactivations rather than infection chains of *de novo *HAdV-A31 infections [14,22]. However, HAdV-A31 may also be transmitted easily in a nosocomial setting between immunosuppressed patients, as high amounts of HAdV-A31 are spread with faeces [23].

In spite of this increasing clinical relevance of HAdV-A31, the virus had not yet been completely sequenced. Therefore, we determined the complete nucleotide sequence of the HAdV-A31 prototype strain in order to search for unique sequence motifs which may be associated with its high virulence. In addition, we compared several virulence associated gene regions (E1A, E3, E4, fiber knob, penton base, protein IX, and pX) of seven clinical isolates and the HAdV-A31 prototype.

## Results

### General properties

**Figure 1:**
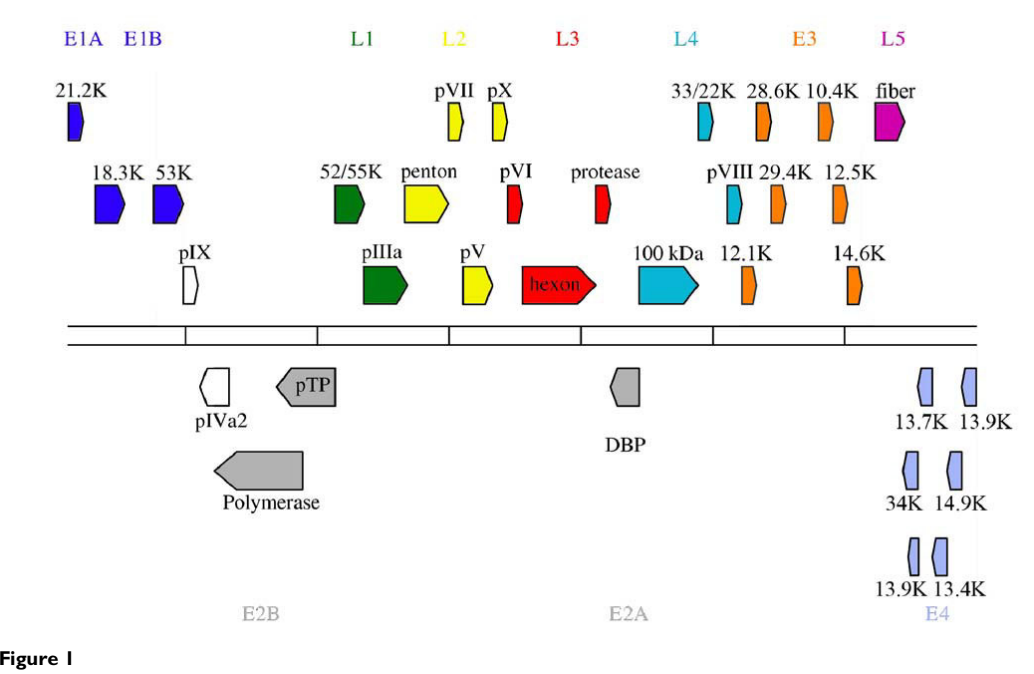
**Map of the genome organization and transcription units of HAdV-A31**. Early and late transcription units are represented in different colors, intermediate gene products in white. The block arrows represent the predicted protein, titled either by protein name or predicted molecular size. Orientation of the arrows indicates the direction of transcription.

The complete genomic sequence of HAdV-A31 is 33,763 base pairs in length and was submitted to GenBank as AM749299. The plus strand has a base composition of 22.89% G, 23.48% C, 27.49% A and 26.14% T. The GC content is 46.36%. As other Mastadenoviruses, HAdV-A31 is organized into four early, one intermediate and five late transcription units. We identified 34 coding regions that are homologue to previously described gene products of other human adenoviruses (Figure 1). The annotation of the predicted coding gene regions is listed in Table 1.

**Table 1:** HadV-A31 annotation of coding regions

Region	Common name	Product	Location	Length in aa
E1A	n.n.	29.4K	479-1045 & 1124-1348	266
E1B	19 K small t-antigen	18.3 K	1488-1955	156
	55 K large t-antigen	53.1 K	1793-3208	472
Intermediate	IX	14.9 K	3290-3721	144
	IVa2	50.9 K	c3757-5093 & c5372-5384	449
E2B	DNA Polymerase	134.6 K	c4866-8042 & c13193-13201	1184
	pTP	73.1 K	c8222-9982 & c13193-13201	633
L1	52/55 K	41.8 K	10307-11413	368
	pIIIa	64.4 K	11435-13171	578
L2	III (Penton Protein)	57.1 K	13246-14763	505
	V	39.8 K	15373-16416	347
	pVII	20.4 K	14777-15340	187
	pX	7.9 K	16440-16658	72
L3	pVI	28.5 K	16734-17516	260
	Hexon	103.7	17575-20343	922
	Protease	23.3 K	20370-20981	203
E2A	DNA binding Protein	55.3 K	c21065-22522	485
L4	100 K	86.8 K	22551-24878	775
	22 K	20.2 K	24616-25146	176
	33 K	23 K	24616-24913 & 25092-25393	199
	pVIII	25.2 K	25452-26153	233
E3	12.5 K	12.1 K	26153-26470	105
	CR 1 alpha	28.6 K	26424-27212	250
	CR 1 beta	29.4 K	27215-27967	262
	RID alpha	10.5 K	27999-28274	91
	RID beta	12.5 K	28271-28600	109
	14.7 K	14.6 K	28593-28979	128
L5	Fiber	58.9 K	29157-30827	556
E4	ORF 1	13.9 K	c33046-33429	127
	ORF 2	14.9 K	c32619-33014	131
	ORF 3	13.4 K	c32272-32622	116
	ORF 4	13.7 K	c31904-32266	120
	ORF 5	33.9 K	c31102-31971	289
	ORF 6/7	13.9 K	c30861-31082 & c31834-31971	119

### Phylogeny

**Figure 2:**
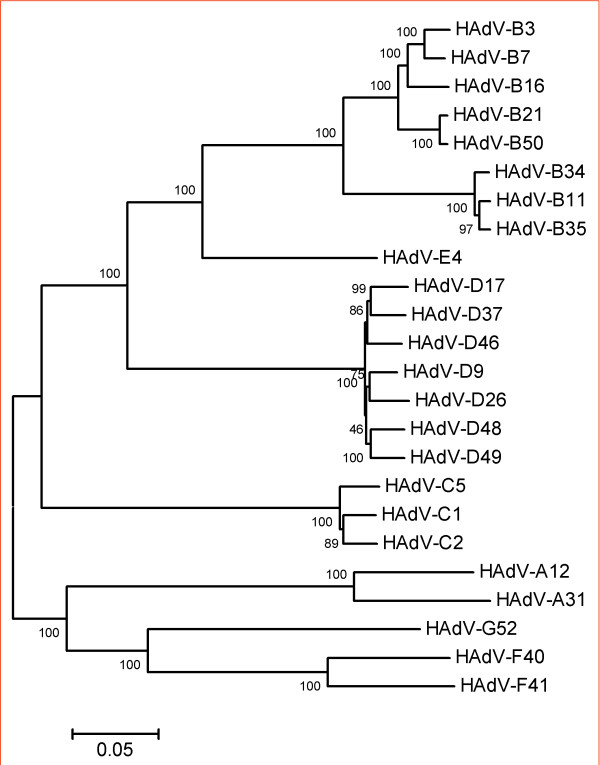
**Phylogenetic analysis of all available complete genomic HAdV sequences representing all human adenovirus species (A to G), including the newly generated HAdV-A31 sequence**. The tree was generated with MEGA 3.1 using neighbor-joining method, bootstrap values (%) were generated with 1,000 pseudoreplicates. For nucleotide accession numbers see Methods section.

Phylogenetic analysis of the whole genomic sequence of HAdV-A31 was performed by using the neighbor-joining method. Representative members of all HAdV species were included in the analysis. HAdV-A31 clustered as expected to the species HAdV-A, close to HAdV-A12 (Figure 2).

**Figure 3:**
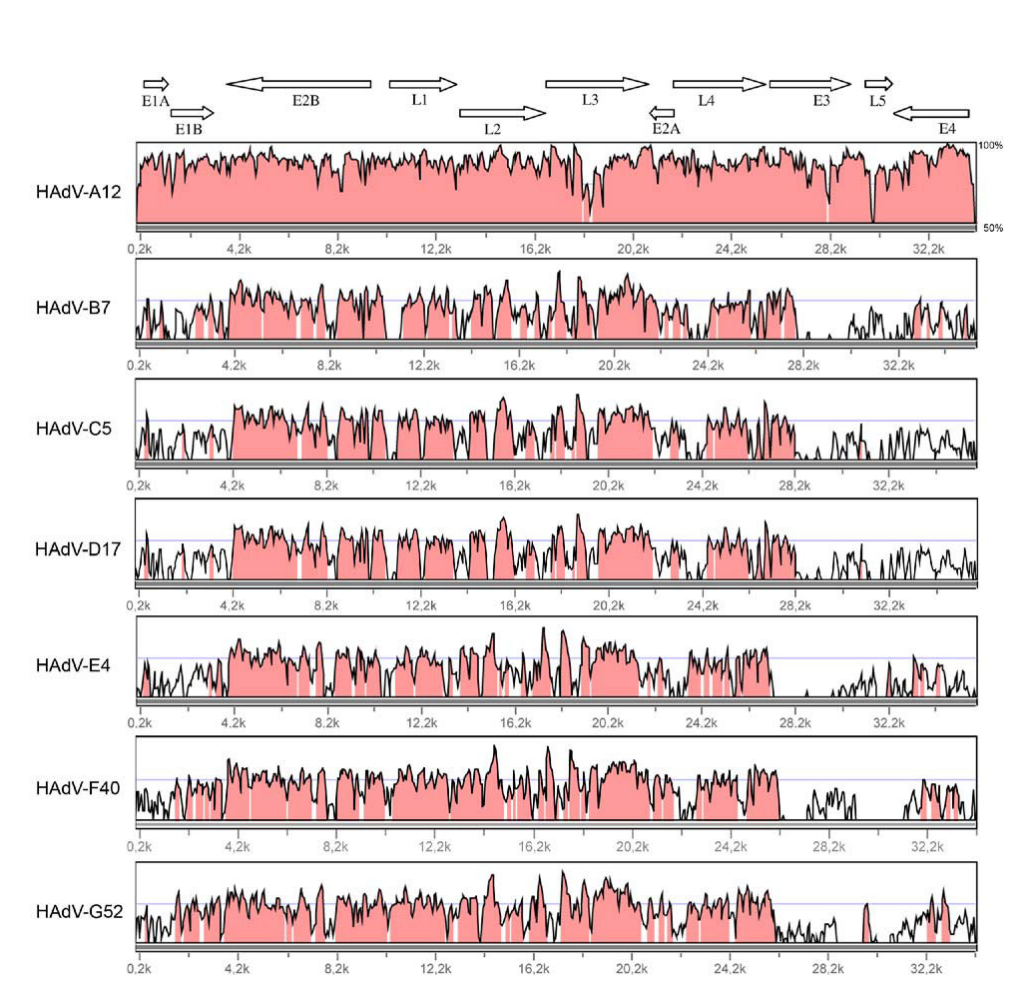
**Global pairwise sequence alignment of the HAdV-A31 genome with representative types of each HAdV species**. The x axis shows the genome position, the y axis shows the sequence conservation in percent. Arrows on top display the transcription units and the direction of their transcription.

A global pairwise alignment was constructed using the mVISTA Limited Area Global Alignment of Nucleotides (LAGAN) [24] in order to compare the predicted whole genomic sequence of HAdV-A31 to the representative types of each species. The graphical alignments showed the close relationship between HAdV-A31 and -A12 with the exception of the coding regions for immunogenic determinants (hexon, fiber). Interestingly, the early transcription unit E3, which is not under selective pressure by the immune system, also showed a significant divergence between HAdV-A31 and HAdV-A12 (Figure 3).

### ITR

HAdV's inverted terminal repeats (ITR) and its flanking DNA regions exhibit several binding sites for viral proteins and a set of cellular factors for efficient adenoviral DNA replication [25]. HAdV-A31's ITRs are 148 bp in length. A nuclear factor III (NF-III) binding site described as conserved among most human adenoviruses as a 5'ATGNNAATGA 3' sequence motif (ATGCAAATAA in HAdV-E4) [26] was not identified in the HAdV-A31 ITR, but a 5'ATGAAGTGGG 3' sequence at position bp 46-55 may function as a binding site for NF-III. HAdV-A12 also lacks the classical NF-III binding motif but has the same 5'ATGAAGTGGG 3' motif as HAdV-A31 at bp 46-55. The conserved NF-I binding site (*5*'TGGACTTGAGCCAA 3') was predicted for HAdV-A31 at position 25-38.

HAdV-A31 ITRs also revealed binding sites for the transcription factors SP1 and ATFs. The binding of ATFs to a 5'TGACGT 3' motif has been shown to be important for efficient viral growth of HAdV-C5 [27]. Whereas all other human adenoviruses (including HAdV-A12) reveal exactly this ATF binding motif, HAdV-31's putative single ATF binding site between bp 113 - 117 showed a C instead of the commonly observed T at the end of the motif. An SP1 binding site was present as a GC-rich region between bp 52 - 61 (5' TGGGCGGAGT 3'). The extreme termini of the ITRs had the common 5'CTATCTATAT 3' motif that is required for viral replication and also protects the viral genome by ORP-A binding from DNAse-I digestion. The core origin of DNA replication, which binds the complex of preterminal protein (pTP) and DNA polymerase, was present in the highly conserved motif 5' ATAATATACC 3' between bp 9 and 18 in HAdV-A31.

Human elongation factor 1-alpha (EF-1A) is known to be an efficient factor for enhancement of E1A gene region transcription. HAdV-A31 revealed only two putative EF-1A binding motifs (5'GCCGGATGT 3'), which are located within the ITR region at bp 105-113 and 136-144, whereas the five EF-1A binding sites are located upstream from the E1A promoter region of HAdV-C5 [28].

### E1A coding region

**E1A **is the first gene expressed after adenoviral infection [3]. The predicted gene product of the HAdV-A31 E1A ORF was 266 amino acids in length with a molecular weight of 29.4 K. Most of the functional sites of E1A proteins are located in conserved regions CR1 - CR4 [29]; homologue amino acid sequences at corresponding positions were found in HAdV-A31. The predicted E1A protein of HAdV-A31 revealed a sequence (EQDENGMAHVSAAAAAAAANRER) at position 122 - 144 that is homologue to the E1A protein of HAdV-A12. The latter was previously identified as a repressor for the presentation of MHC class I molecules on the surface of infected cells. Essential for this function of HAdV-A12 is a stretch of 23 amino acids, which could not be found in the E1A proteins of other HAdV species [30].

HAdV-A31 revealed the essential Rb-protein binding motif as a LLCYE sequence at residues 107-111. A C-terminal binding protein (CtBP) interacting motif (PVDLS), which is probably equivalent to PLDLS in other HAdV types, was found near the C-terminus of the E1A protein. Two zinc finger motifs CSLC and CKSC, both essential for transactivation of transcription [31] are located in the CR3 region.

### E1B coding region

Two ORF in the **E1B **gene region coded for predicted proteins of 18.3 K and 53.1 K molecular weight. The predicted 18.3 K protein was 156 amino acids in length and homologue to the small ***t***-antigen, which is known to have anti-apoptotic features [32]. While the stretch of the first 145 amino acids of the HAdV-A31 E1B 18.3 K protein shared a homology of 93.1% to the corresponding gene product of HAdV-A12, the C-terminus is highly divergent (only 27.7% identity).

The second E1B gene product was a predicted 53.1 K protein, 472 amino acids in length and homologue to the large ***t***-antigen. It defends the virus against the p53 mediated antiviral host cell response by binding to the DNA linked p53 protein directly and repressing its function as a transcriptional activator. In addition to this direct interaction, a complex comprising the large ***t***-antigen, the E4 ORF 6 gene product and a set of cellular cofactors build an E3-ligase-complex that also degrades the p53 protein [33,34]. Essential for the stability of this complex is a BC-Box binding motif ((A,P,S,T)LxxxCxxx(A,I,L,V)), present in the predicted E1B 53 K gene product of HAdV-A31 as an ALRPDCTYKI motif at amino acid position 156-164.

Amino acid comparison with the predicted large ***t***-antigen of HAdV-A12 revealed a high sequence divergence (only 55.5% identity) between residues 38 - 100. However, the C-terminus of large ***t***-antigen, which was described as essential for repressing the function of p53 in HAdV-A12 infected cells, showed a high sequence homology to HAdV-A12.

### E2 coding region

E2 is divided into two transcriptional units, E2A and E2B. Transcription of the E2 region is controlled by a well characterized RNA polymerase II promoter (major promoter) on the complementary strand, which is transactivated by E1A and E4 ORF6/7 gene products. The E2 promoter region of HAdV-A31 was located between bp c25299-c25431. It lacked at least one of the typical E2F (bp c25422-25429, TTTCCCGC) binding motifs as described for HAdV-C2 and -C5. However, a putative binding motif for E4F1 (base pair c25430-c25436, ACGTCAC) was predicted. E4F1 is another cellular transcription factor that stimulates the transcription of E4 genes mediated by E1A gene products [35]. Moreover, the E2 promoter of HAdV-A31 had a binding motif for ATF (bp c25431 - c25439, TGACGTCAC) and a TATA-like sequence for binding TBP (bp c25381-c25386, TTAAGC). Within the **E2A **region an ORF for a predicted protein of 55.3 K was identified. The product was homologue to the DNA binding protein (DBP) and revealed a zinc-binding domain at amino acid position 229-242 (HxC8CxH).

The **E2B **region of HAdV-A31 encoded a predicted 134.6 K protein homologue to the HAdV DNA polymerase protein, and a 73.1 K gene product homologue to the precursor of the terminal protein (pTP), both on the complementary strand. The common nuclear localization signal of pTP was present as RLPVRRRRRRLP motif at amino acid position 351-362.

### E3 coding region

The **E3 **region is known to code for a set of proteins that are not essential for virus replication *in vitro *but are important factors for interfering with the host immune response [36,37]. Six ORFs were identified in the E3 transcription unit of HAdV-A31, encoding putative gene products of the following sizes: 12.1 K, 28.6 K, 29.4 K, 10.5 K, 12.5 K, and 14.6 K. The organization is similar to the E3 region of the closely related HAdV-A12 [38].

The 12.1 K predicted protein was homologue to a 12.5 K protein which is present in all HAdV types beside the enteric HAdV-F40 and -F41. The sequence identity between HAdV-A31 and -A12 was comparatively high (96.1%).

**Figure 4:**
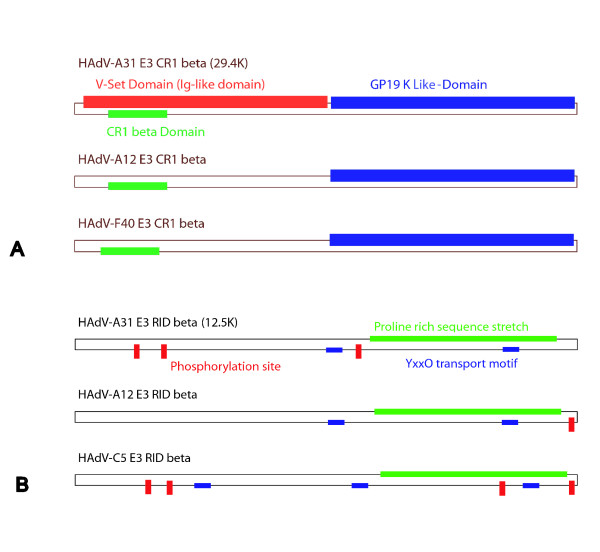
**Schematic view of the predicted E3 CR1 beta (A) and the E3 RID beta (B) proteins of HAdV-A31, -A12 and -F40 or -C5, respectively**. (A): a V-Set domain (red box) was only predicted within the N-terminal region of the E3 CR1 beta protein of HAdV-A31. (B): N-terminal phosphorylation sites (small red boxes) were predicted both for the HAdV-A31 and -C5 RID beta proteins, but not for HAdV-A12. Domain predictions were carried out using web based Pfam, ProSite and BLASTp.

The second and third reading frames, coding for a 28.6 K (CR1 alpha) and a 29.4 K (CR1 beta) protein, revealed comparatively low identities of 76.1% and 72.1% to homologue E3 gene products of HAdV-A12. CR1 alpha and beta were described as species HAdV-A specific gene products [39]. Prediction of transmembrane domains suggested that both gene products were type Ia transmembrane proteins. Protein Blast search of CR1 beta showed homologies between the putative gene product of HAdV-A31 and proteins of the SLAM (signalling lymphocyte activation molecule) family. Patterns of predicted functional domains of HAdV-A31 in comparison to HAdV-A12 and -F40 are shown in Figure 4A.

The 10.5 K and 12.5 K ORF of HAdV-A31 were identified to be homologue to the known RID (receptor internalization and degradation) alpha and beta proteins, which are present in the E3 transcription units of all HAdV. Both proteins are non-covalently associated integral membrane proteins [40]. The N-terminal phosphorylation sites pattern of HAdV-A31 RID-beta was more similar to HAdV-C5, whereas HAdV-A12 lacked these phosphorylation sites completely (Figure 4B). YxxO motifs and proline rich sequence stretches near the C-terminus, which are conserved among all HAdV and may be part of a protein interacting domain [39], were identified in the predicted RID beta protein of HAdV-A31 (Figure 4B). YxxO motifs function as signals for transport and internalization into lysosomes/endosomes.

RID alpha is a hydrophobic protein and appears in two isoforms [39]. Depending on cleavage of the signal peptide, it either functions as a type I or a type II transmembrane protein. An analogue cleavage pattern was predicted for the RID alpha protein of HAdV-A31 by using the web based TMHMM v. 2.0 software. As described for other human adenoviruses dileucine, dileucine-like and YxxO motifs are also present in the cytoplasmatic portion of the HAdV-A31 RID alpha gene product.

The last ORF of the E3 region encodes a 14.6 K protein that is homologue with a sequence identity of 88.2% to the 14.7 K protein of HAdV-A12. A corresponding protein is present in all species of HAdV species [41]. It has been shown to be located in the cytosol and nucleolus, functioning as an inhibitor of TNF mediated cell lysis. Structure and function analysis of the 14.7 K of HAdV-C5 indicate that its biological function does not depend on single conserved subdomains but that critical amino acids are distributed throughout the entire protein. It has been shown that a set of three cysteine residues between amino acid 40 and amino acid 120 are essential for the function of the protein [36,37]. Corresponding cysteine residues were identified in the predicted 14.6 K protein of HAdV-A31.

### E4 coding region

Transcription of the E4 region on the complementary strand is stimulated by the E1A gene product and the cellular transcription factor E4F1. An E4F1 binding motif ACGTCAC (of the consensus sequence ACGTMAC) was identified in HAdV-A31 at bp c33513-c33519 located upstream of the putative TATA box at bp c33479-c33502.

Corresponding to the E4 transcription units of other human adenoviruses (including the closely related HAdV-A12), six ORFs were predicted within the genome of HAdV-A31. These ORFs encoded for respective proteins of 13.9 K, 14.9 K, 13.4 K, 13.7 K, 33.9 K and 13.9 K (spliced gene product ORF6/7). The predicted gene product of the HAdV-A31 ORF6/7 exhibited only a single BC-Box like motif (SAWPECNSLT) slightly different to the consensus motif sequence (A,P,S,T)LxxxCxxx(A,I,L,V). This is in contrast to HAdV-C5, which shows two BC-Box motifs that are both required for degradation of p53 [34].

### Virus associated RNA

Sequence coding for the VA RNA was predicted by comparison with HAdV-A12 and is located at bp10146 - 10286 in the genome of HAdV-A31.

### Intermediate genes

**Figure 5:**
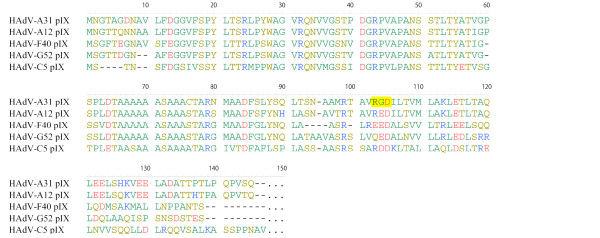
**Multiple alignment of the protein IX amino acid sequences of HAdV-A31, -A12, -C5, -F40 and -G52**. The RGD motif found in the protein IX of HAdV-A31 is highlighted.

Two proteins are encoded in the adenoviral intermediate gene region, IX and IVa2. The protein IX of HAdV-A31 was predicted as a 14.9 K protein of 144 amino acids in length. It is a structural component of the virus and influences hexon-hexon interaction. A stretch of 32 amino acids is conserved among all HAdVs and is supposed to be crucial for the incorporation of the protein IX into the capsid. Corresponding amino acids were identified in the protein IX of HAdV-A31 at position 14-45. Surprisingly, we identified an RGD motif in the amino acid sequence of HAdV-A31 protein IX at amino acid position 102-104 (Figure 5). This RGD motif is only 40 amino acids distant from the C-terminus that is assumed to be exposed on the surface of the virus [42]. Moreover, the RGD motif is located at the N- terminal end of a coiled coil region predicted by the COILS web based software [43]. This prediction suggested that the RGD motif is possibly solvent exposed and may be functional in binding to alpha integrins.

The IVa2 ORF is transcribed from the complementary strand and codes for a 50.9 K protein of 449 amino acids in length. The protein IVa2 of human adenoviruses binds the A-repeat sequences at the left end of the genome and is involved in the process of viral DNA packaging and virus assembly. Furthermore, it is assumed to have a function as a transcriptional activator of the late adenoviral genes [44].

### Late genes

The major late transcription unit (MLTU) encodes the majority of the virus structural proteins and is organized into five subregions L1-L5. The initiation of late gene transcription is controlled by a promoter region that is present in all human adenoviruses and termed as major late promoter (MLP) [3]. Based on sequence comparison, the putative inverted CAAT box (TGATTGGTT) was identified at bp 5633-5641 and the TATA box at position 5684-5690 in the genome of HAdV-A31. The **L1 **transcription unit encodes the 52/55 K and the pIIIa protein. An ORF coding for a 41.8 K protein as a homologue of 52/55 K that is known to be functionally relevant in the process of virion assembly was predicted. A second ORF encodes the putative pIIIa protein of 64.4 K that is associated with the hexon protein and present on the outer surface of the virion.

Four ORFs within **the L2 **region were identified: coding for the penton base protein (III), proteins V, pVII and the pX. The predicted penton base protein of HAdV-A31 is 505 amino acids in length and exhibits an integrin (α_v_β_3 _and α_v_β_5_) binding RGD motif at amino acid position 301-303. In addition to the RGD sequence, an LDV motif was present at amino acid position 285-287 of HadV-A31. LDV motifs were described as interacting with another group of integrins (α_4_β_1 _and α_4_β_7_) [45-47].

The predicted homologue of protein V has a molecular weight of 39.8 K and is 347 amino acids in length. A 20.4 K gene product is homologue to the pVII, and a predicted product of 7.9 K represents the pX protein of HAdV-A31. All three proteins are described as being core proteins and are associated with the virus DNA [48]. The sequence generated for pX protein of HAdV-A31 was highly divergent from the HAdV-A31 pX sequence previously available in GenBank [GenBank: U14653] (nucleic acid sequence homology 70.4%, amino acid 53.4%). Therefore, this region was re-sequenced a second time and additionally sequenced for all seven clinical isolates; our previous result was confirmed. Most likely, the databank sequence relates to a subtype of HAdV-A31 or is mislabelled.

**The L3 **transcription unit encodes three proteins; we could identify ORF for pVI, the hexon protein (II) and the virus encoded protease. The predicted pVI protein of HAdV-A31 is 260 amino acids in length and has a molecular weight of 28.5 K. It revealed two nuclear localization signals (KRPRP at amino acid 136-140 and KRRR at amino acid 255-258) close to its C-terminus. Corresponding motifs of HAdV-C2 play an important role in directing cytoplasmatic proteins to the nucleolus and thus might be functionally active as nuclear localization signals [49]. The mature pVI protein is a minor capsid component.

The predicted hexon protein of HAdV-A31 has a molecular weight of 103.7 K and a length of 922 amino acids. Due to its serotype defining main neutralization determinant ε, it was highly divergent from the closely related HAdV-A12.

The predicted ORFs of the **L4 **region of HAdV-A31 encode putative proteins of 86.8 K (as equivalent to100 K), 20.2 K (as equivalent to 22 K), 23 K (as equivalent to 33 K), 25.2 K (as equivalent to pVIII), respectively.

Only one ORF is present in the **L5 **region of HAdV-A31, encoding a 58.9 K protein of 556 amino acids in length, representing the fiber protein (IV), a major structural protein with the highly variable hemagglutination determinant in its terminal knob structure. The fiber shaft was found to be 31 amino acids (two pseudorepeats) shorter than the fiber shaft of HAdV-A12. We detected a deletion in the 3^rd ^non-consensus β-repeat of HAdV-A31 fiber shaft and a KLGXGHXFS motif in the penultimate repeat instead of the classical KLGXGLXFD/N flexibility consensus motif of other adenovirus serotypes. This could affect the function of the flexibility regions and influence the cell attachment.

### Comparison of clinical isolates to the prototype sequence

The coding regions of the E1A, E3, E4, penton base, fiber knob, protein IX and pX genes of seven clinical isolates were sequenced and compared. All strains have been isolated from paediatric patients with disseminated infections following hSCT. Overall, the pX, IX, E3 RID alpha, E4 ORF 3 and ORF 4 nucleic acid sequences of all isolates were 100% identical to the prototype. The lowest amino acid identity was observed in the E3 CR1 beta gene with 99.2%. In Table 2, gene product and amino acid substitutions of clinical strains are listed. Lowest S/N ratios were calculated for the E3 CR1 beta (2.36), whereas only synonymous mutations were observed in the penton base protein of several clinical isolates. Two isolates revealed an amino acid substitution in the penton protein at position 305 (tyrosine residue instead of phenylalanine), which is close to the functional RGD motif and may influence integrin binding. Interestingly, all wild type strains revealed the additional RGD motif in protein IX of the HAdV-A31 prototype strain. All motifs described for the HAdV-A31 prototype strain were conserved in the clinical isolates.

**Table 2:** Sequence comparison of clinical isolates to the HAdV-A31 prototype sequence

Clinical isolate	V04-03789	0105019310	2006001610	95/8866	95/6956	96/783	95/6315
Origin	Regensburg, Germany	Hannover, Germany	Hannover, Germany	Nancy, France	Nancy, France	Nancy, France	Nancy, France
Year	2004	2001	2006	1995	1995	1996	1995
E1A	S56C, V222N (5)						
E3 12.1		E34Q (1)					A5T (3)
E3 CR1 α		Q71E, N159S (9)					
E3 CR1 β	Q164E, T242I (2)	T242I (7)	Q164E, T242I (3)	D130N, Q164E (2)	Q164E (3)	Q164E, T242I (4)	Q164E, T242I (2)
E3 RID β	M13V, E107G (2)	E107G (1)	E107G (1)		E107G (1)	E107G (1)	E107G (1)
E3 14.7	T106N (3)	T74A, T106N (4)	T106N (4)		T106N (5)	T106N (5)	T106N (3)
PB		F259Y (7)			F259Y(8)		
FK	V40L, R62Q (4)	R62Q (4)	R62Q (3)		R62Q (3)	R62Q (3)	V40L, T53A, R62Q (5)
E4 ORF1	P119T (1)			V59L (2)			
E4 ORF2	L84P (1)						
E4 ORF5	R51K (6)						
E4 ORF6/7				K105N, I115S (3)			

The coding regions which showed amino acid substitutions in at least one isolate are listed. For each isolate, the sequence positions of amino acid substitution are noted. Additionally, the number of mutations in the nucleotide sequence is indicated in brackets.

## Discussion

We determined the complete 33,763 base pair genome of the HAdV-A31 prototype strain and identified 34 putative genes. HAdV-A31 is a highly significant pathogen, which has been frequently isolated from severely affected hSCT recipients, and frequently presents itself as a disseminated disease, which is usually caused by species HAdV-C [10]. Experimental data strongly suggested that species HAdV-C types have the ability to establish latent infections in mucosal lymphocytes and that stimulation of those cells can cause viral reactivation in cases of immunosuppression [21]. Especially the early gene products (e.g. E3), which counteract host anti-viral defence mechanisms, might play a key role in the process of persistence and reactivation [39,50,51]. A similar mechanism of persistence and reactivation can be suspected in case of HAdV-A31, which would explain its high incidence in immunosuppressed patients. Therefore, the early coding regions E1A, E1B and E3 of the newly generated HAdV-A31 prototype sequence were analyzed in detail for functional motifs. Moreover, these genome regions of seven HAdV-A31 wild type strains isolated from immunosuppressed patients were also sequenced in order to clarify whether a highly pathogenic subtype of HAdV-A31 was circulating in recent years. However, analysis of nucleic acid and predicted amino acid sequences of seven HAdV-A31 clinical isolates revealed high identity to the prototype strain (Table 2). Non-synonymous mutations in clinical isolates clustered in the E3 region, but did not affect previously described and predicted functional sites and motifs (Figure 4) [37,39]. An S/N ratio of 2.36 within the species HAdV-A specific CR1 beta protein suggested selection of a potentially highly functional E3 protein, which is assumed to interact with the immunosurveillance of adenovirus infected cells [39]. Unfortunately, experimental data about the functions of the E3 gene products CR1 alpha and beta were not available. Therefore, *in silico *protein analysis of the predicted E3 CR1 alpha and beta proteins of HAdV-A31 were performed. Interestingly, the E3 CR1 beta protein of the HAdV-A31 prototype and all analyzed clinical isolates were predicted to exhibit an immunoglobulin-like (V-set) domain, which was predicted neither for the closely related HAdV-A12 nor for the corresponding E3 gene products of the related enteric species F adenoviruses. Immunoglobulin-like domains are described to be involved in cell-cell interaction of the immune system [52]. A similar domain was previously described as involved in a novel feature of the soluble 49 K E3 gene product of HAdV-D19a adenoviruses [37]. Functional studies of the 49 K protein of HAdV-D19a demonstrated proteolytic processing and secretion of the type Ia transmembrane protein [37]. Furthermore, a NK cell binding activity was detected and the immunoglobulin-like domain of the HAdV-D19a 49 K protein was assumed to interact directly with NK cells, protecting infected cells against lyses [37]. For comparison, *in silico *protein analysis of HAdV-A31 CR1 beta also predicted a type Ia transmembrane domain, a (signalpeptide-)cleavage probability of ~83%, a C-terminal sorting motif and various glycosylation sites, all of which are analogous to the confirmed predictions for HAdV-D19a E3 49 K protein.

In contrast to these predictions for CR1 beta, potential functionality of the predicted CR1 alpha protein of HAdV-A31 have remained obscure, since all performed analyses and predictions did not reveal similarities with functional sites or motifs of any E3 counterparts of other HAdV species. Overall, CR1 alpha and beta amino acid sequence comparison between HAdV-A31 and -A12 showed a particularly low identity of only 76.1% and 72.1%, respectively. This is in considerable contrast to corresponding E3 gene products of HAdV-F40 and -41, which had a high intraspecies homology of 98.8% and 99.2%, respectively. Significant differences between both species A adenoviruses were also identified in the theoretical molecular weight and isoelectric point (pI) for CR1 alpha and beta gene products computed and visualized in virtual 2D gel analysis (data not shown). These considerable differences between HAdV-A31 and -A12 in the primary structure, protein size, pI and predicted functional domains consequently indicated differences in protein function and might be an important feature in explaining the described higher virulence of HAdV-A31.

Comparison of the other E3 gene products, 12.5 K, RID alpha, RID beta and 14.7 K, revealed sequence identities between 80 and 96% with HAdV-A12. With the exception of predicted phosphorylation sites of RID beta (Figure 4B), previously described functional sites and motifs are conserved in HAdV-A31 and HAdV-A12, suggesting a comparable functionality. As determined for HAdV-A12 and species F adenoviruses, the E3 transcription unit of HAdV-A31 also lacked the extensively studied GP 19 K protein, which down regulates the expression of MHC I molecules and NK activation receptors [53-55]. As a substitute for this important immune escape mechanism, the E1A gene product of HAdV-A12 was identified to down regulate the expression of MHC class I molecules by interfering with the transcription of MHC I gene products [56]. This unique feature can be assumed for the E1A protein of HAdV-A31 as well, because amino acid stretches of HAdV-A12, which have been identified as essential for this mechanism, were identified in the predicted E1A gene product of HAdV-A31 at corresponding positions. While the E1A protein is known to have pro-apoptotic features, the E1B 19 K gene product of HAdV-C5 shares homology with the cellular anti-apoptotic Bcl-2 protein and interferes with a set of different cellular pro-apoptotic proteins (Bak, Bax and Nbk/Bik), thus protecting infected cells against apoptosis [32]. Comparison of the E1B 19 K small ***t***- antigen homologue of HAdV-A31 with HAdV-A12 revealed high divergences of the C-terminus. Since the E1B 19 K C-terminus of HAdV-C5 has been identified as exhibiting a functional domain that influences the lateral viral spread of HAdV-C5 by interfering with cellular apoptotic pathways [57], the observed divergence between HAdV-A31 and -A12 might have functional relevance.

In addition to the capability of persistence and of reactivation by interfering with the host immune response, the capacity for dissemination seems to be essential for a highly pathogenic HAdV subtype. For example, an outbreak of the strictly enterotropic HAdV-F41 did not cause any fatalities in paediatric hSCT recipients [17], which might be due to the missing integrin binding RGD motif within the penton protein of species F adenoviruses. Since fatal dissemination of adenoviruses affects various organic systems, a more promiscuous behaviour in cell attachment and entry can be assumed to be favourable [58]. Interestingly, the protein IX of HAdV-A31 revealed an RGD motif at amino acid position 102 - 106, which is conserved within all seven analyzed clinical isolates. This RGD motif is only 40 residues distant from the C - terminus, and it is unique among all sequenced human adenoviruses. As described previously, the C-terminus of HAdV protein IX is exposed on the outer surface of the virion [42,59]. It was shown for protein IX of BAdV-3, which is 125 amino acids in length that the N-terminus (13 - 32) and the central region (61 - 80) have immunogenic sites but are not exposed on the outer surface of the virion [60]. So far, no structural data about the exposed region of the C-terminus of protein IX of HAdV-A31 is available, but the observations for BAdV-3 indicate that the amino acid stretch of the protein IX of HAdV-A31 that contains the RGD motif might be present on the outer surface of the virion. As the HAdV-A31 fiber shaft is shorter and probably less flexible than the fiber shaft of HAdV-A12, a functional binding of the secondary cellular receptor to the RGD motif of protein IX instead of the RGD motif in the penton base may be possible or even preferred. Moreover, the construction of adenoviral vectors with an incorporated RGD motif within the C - terminus of protein IX has demonstrated that the additional RGD motif resulted in a significant augmentation of fiber independent infection of CAR-deficient cell types [60]. Therefore, the additional RGD motif within the protein IX HAdV-A31 might lead to a more effective targeting and internalization, and could be a factor in increased transmission and infectivity of the virus.

In addition to protein IX, sequence comparison of the cellular receptor binding sites of the penton protein of the clinical isolates with the HAdV-A31 prototype revealed an amino acid substitution (F305Y) in two clinical isolates. This substitution is close to the functional RGD motif and may influence integrin binding. Moreover, a low S/N ratio of 2.78 (with exception of clinical isolate number: 95/8866) for the fiber knob indicated selective pressure on a major structural protein of the clinical isolates.

Overall, the sequence divergence of the isolated clinical strains in comparison to the prototype sequence was determined to range between 99.2 and 100%. These results suggested that all isolated strains were closely related to the prototype; a single outbreak subtype associated with severe disease in stem cell transplant recipients was not identified (Table 2). This is in congruence with previous results of RFLP analysis of 79 HAdV-A31 wild type isolates from immunocompromised and immunocompetent hosts, where a wide variety of slightly genetically different subtypes of HAdV-A31 was described [22].

## Conclusion

Overall, studying the HAdV-A31 prototype seemed to be sufficient to elucidate the high incidence and disease burden in immunosuppressed patients because HAdV-A31 strains recently circulating in immunocompromised patients were closely related to the prototype. Unique motifs of the HAdV-A31 E1 and E3 regions may provide immune modulation and perhaps virus persistence. The additional RGD motif of protein IX may promote promiscuous tropism for various tissues and enhance dissemination. Further studies of its biological relevance *in vivo*/*in vitro *are necessary to clarify its potentially unique characteristics.

## Methods

### Sequencing strategy

Previously published partial nucleotide sequences of HAdV-A31 and the genomic HAdV-A12 sequence were used to design the PCR primer for the production of genome fragments of the HAdV-A31 up to 5000 base pairs in length. Depending on the size of the amplicon, these fragments were either cloned and sequenced subsequently or sequenced directly from the amplicon. Both strands were sequenced by primer walking with overlapping sequencing reactions. Furthermore, we resequenced the HAdV-A31 sequences already available in GenBank (with exception of the hexon gene [61]). On the basis of the newly generated complete nucleotide sequence of HAdV-A31, we designed the PCR and sequencing primers for sequencing the E1A, E3, E4, fiber knob and penton base coding regions of seven clinical HAdV-A31 isolates.

### HAdV prototype strain, wild type strains and cells

The HAdV-A31 (ATTC) prototype strain was obtained from the American Type Culture Collection (ATCC). Wild type strains of HAdV-A31 had been isolated between 1995 - 2004 from cases of severe HAdV disease in France and Germany (Table 2) [14].

All viruses were propagated on the human lung cancer cell line A549 (ATCC, CCL-185) on 75-cm^2 ^or 25 cm^2 ^culture flasks using DMEM medium with 5% of FBS (Biowest, Nuaillé, France) and 1% Penicillin/Streptomycin (Cytogen, Sinn, Germany) added. When the cytopathogenic effect was above 50%, cells were washed with PBS^- ^(Cytogen) and lysed using Trypsin/EDTA (Cytogen). The DNA was extracted with the Qiagen blood kit (Qiagen, Hilden, Germany).

### PCR amplification and cloning of DNA fragments

For the amplification of fragments up to 500 bp we used the HotStar Mix (Qiagen, Hilden, Germany) in a total volume of 50 μl with 1 μM of each primer and 5 μl of the purified genomic HAdV-A31 DNA. The PCR program began with the activation of the "hot start" DNA polymerase for 15 min at 95°C, followed by 40 cycles consisting of denaturation at 94°C for 20 s, a primer annealing temperature between 52 - 56°C depending on the composition of the used pair of primers for 20 s and elongation at 72°C for 40 s, followed by a final extension step of 72°C for 5 min. All fragments of larger size were amplified using the Expand High Fidelity PCR System from Roche Applied Science (Mannheim, Germany) which achieves a higher transcriptional fidelity by a proofreading activity. PCR reactions were performed in a total volume of 50 μl with 1 μM of each primer and 5 μl of the purified genomic DNA. The program starts with an initial denaturation step at a temperature of 94°C for 2 min, followed by 10 cycles consisting of denaturation at 94°C for 15 s, annealing, depending on the used primer pair, at temperatures between 52 - 56°C for 30 s and an elongation at 68°C for 1 - 4 min depending on the fragment length. These cycles were followed by 20 cycles with the same conditions but with 5 s extension of the elongation phase for each successive cycle. All PCR reactions were performed in a T-personal 48 thermocycler (Biometra, Goettingen, Germany).

### Gel electrophoresis

PCR products were separated in a 1% agarose gel for 60 min at 120 V. DNA extraction from the agarose gels was performed with the Qiagen gel extraction kit according to the manufacturer's recommendations.

### Cloning of PCR products

Depending on their length, extracted DNA fragments were either cloned before sequencing or they were sequenced directly. We performed AT-cloning reactions using the pGEM - T Easy Vector System I (Promega Corporation, Madison, WI, USA) and transfected replication competent E.coli bacteria. Plasmid DNA was extracted using the Qiagen mini prep kit according to the manufacturer's recommendations.

### Sequencing

Cycle sequencing of both DNA strands was performed with rhodamine-labeled dideoxynucleotide chain terminator (DNA sequencing kit; ABI, Warrington, England) and analyzed on an ABI Prism 310 automatic sequencer (Applied Biosystems, Foster City, CA, USA). PCR primers were used for the sequencing reactions.

### DNA and protein analysis

Sequence assembly was carried out with the program SeqMan 5.00 from the DNASTAR software package. DNA and protein homology searches were performed using the NCBI BLAST program. DNA and protein sequence alignment were carried out using the ClustalW algorithm implemented in the BioEdit package (version 7.0.4.1). Genome annotation, analysis of non coding DNA motifs and functional protein motifs were performed by using the web based gene prediction software GENEMARK http://exon.biology.gatech.edu, the DNASIS MAX 2.00.002.002 software and by sequence comparison.

Phylogenetic analysis was performed with the MEGA software package (version 3.1). The phylogenetic trees were constructed with the neighbor-joining method. Bootstrap analysis was performed with 1,000 pseudoreplicates. The graphical alignments were performed with LAGAN 2.0 software http://lagan.stanford.edu/lagan_web/index.shtml[24].

*In silico *protein analysis and motif prediction were performed with the web based Pfam http://pfam.sanger.ac.uk[62] and proSITE http://www.expasy.ch/prosite software [63]. Transmembrane domains were predicted with the TMHMM software http://www.cbs.dtu.dk/services/TMHMM[64]. Virtual 2D Gel analysis was carried out using the JVirGel Version 2.0 software http://www.jvirgel.de[65,66]. Prediction of coiled coil regions was performed using the COILS software http://www.ch.embnet.org/software/COILS_form.html[43].

### Nucleotide sequence accession numbers

HAdV-1 [GenBank: AF534906], HAdV-2 [GenBank: NC_001405], HAdV-3 [GenBank: DQ086466], HAdV-4 [GenBank: AY599837], HAdV-5 [GenBank: AC_000008], HAdV-7 [GenBank: AC_000018], HAdV-8 [GenBank: AB448768], HAdV-9 [GenBank: AJ854486], HAdV-11 [GenBank: AC_000015], HAdV-12 [GenBank: AC_000005], HAdV-16 [GenBank: AY601636], HAdV-17 [GenBank: AC_000006], HAdV-19 [GenBank: AB448771], HAdV-21 [GenBank: AY601633], HAdV-22 [GenBank: FJ404771], HAdV-26 [GenBank: EF153474], HAdV-34 [GenBank: AY737797], HAdV-35 [GenBank: AY128640], HAdV-37 [GenBank: DQ900900], HAdV-40 [GenBank: L19443], HAdV-41 [GenBank: DQ315364], HAdV-46 [GenBank: AY875648], HAdV-48 [GenBank: EF153473], HAdV-49 [GenBank: DQ393829], HAdV-50 [GenBank: AY737798], HAdV-52 [GenBank: DQ923122].

The previously sequenced HAdV-31 hexon protein's accession number is GenBank: DQ149611, pX is GenBank: U14653.

## Authors' contributions

SH carried out the laboratory work, molecular genetic studies, genome annotation, bioinformatic analysis and drafted the manuscript. IM participated in the design of the study and performed the phylogenetic analysis. SD participated in sequencing and analysis of the fiber gene regions. FR participated in sequencing and bioinformatic analysis. AH conceived the study, and participated in its design and coordination and helped to draft the manuscript. All authors read and approved the final manuscript.
